# Chromoblastomycosis

**DOI:** 10.4269/ajtmh.26-0396

**Published:** 2026-09-29

**Authors:** Rahul Mazumder, Apoorva Sharma, Tarun Narang

**Affiliations:** Department of Dermatology, Venereology and Leprology, Postgraduate Institute of Medical Education and Research, Chandigarh, India

A 42-year-old female with no prior comorbidities presented with chief complaint of a painless, slowly enlarging plaque over the dorsal aspect of the forearm for the last 6 months. She denied history of trauma and had no other systemic complaints. Mucocutaneous examination revealed a well-defined 5 × 8-cm hyperkeratotic plaque with a mamillated surface and a few areas showing the presence of black dots and yellowish crusting on the extensor aspect of the left forearm (Figure 1A). The plaque was surrounded by an erythematous border and one smaller plaque sharing an erythematous edge. Dermoscopic examination revealed heavy keratinization with numerous clustered black–red dots across the surface, corresponding to transepidermal elimination of dematiaceous fungi (Figure 1B). Multiple dotted and glomerular vessels were visible at the periphery; some formed lacunae within the hyperkeratosis, with interspersed white lines corresponding to dermal fibrosis. Histopathological examination revealed epidermal hyperkeratosis, parakeratosis, and pseudoepitheliomatous hyperplasia with dense inflammatory infiltrate composed of lymphocytes, neutrophils, and plasma cells with formation of occasional suppurative granulomas. Within these granulomas, clusters of rounded, pigmented, fungal profiles with copper penny appearance were noted (Figure 1C and D). Fungal culture using Sabouraud dextrose agar medium at 25°C revealed brown–black colonies with velvety surface after 3 weeks of incubation, and lactophenol cotton blue mount revealed branching conidiophores bearing single-celled conidia. *Fonsecaea pedrosoi* was isolated from the culture. The patient was subsequently started on oral itraconazole 200 mg twice daily with the diagnosis of chromoblastomycosis. Treatment was intended to be continued for 12 months or longer depending on the clinical response. The patient was not able to come for her 3-month follow-up owing to personal reasons as communicated by a follow-up telephone call but reported regression in lesion size as early as 6 weeks after starting therapy.

**Figure 1.**
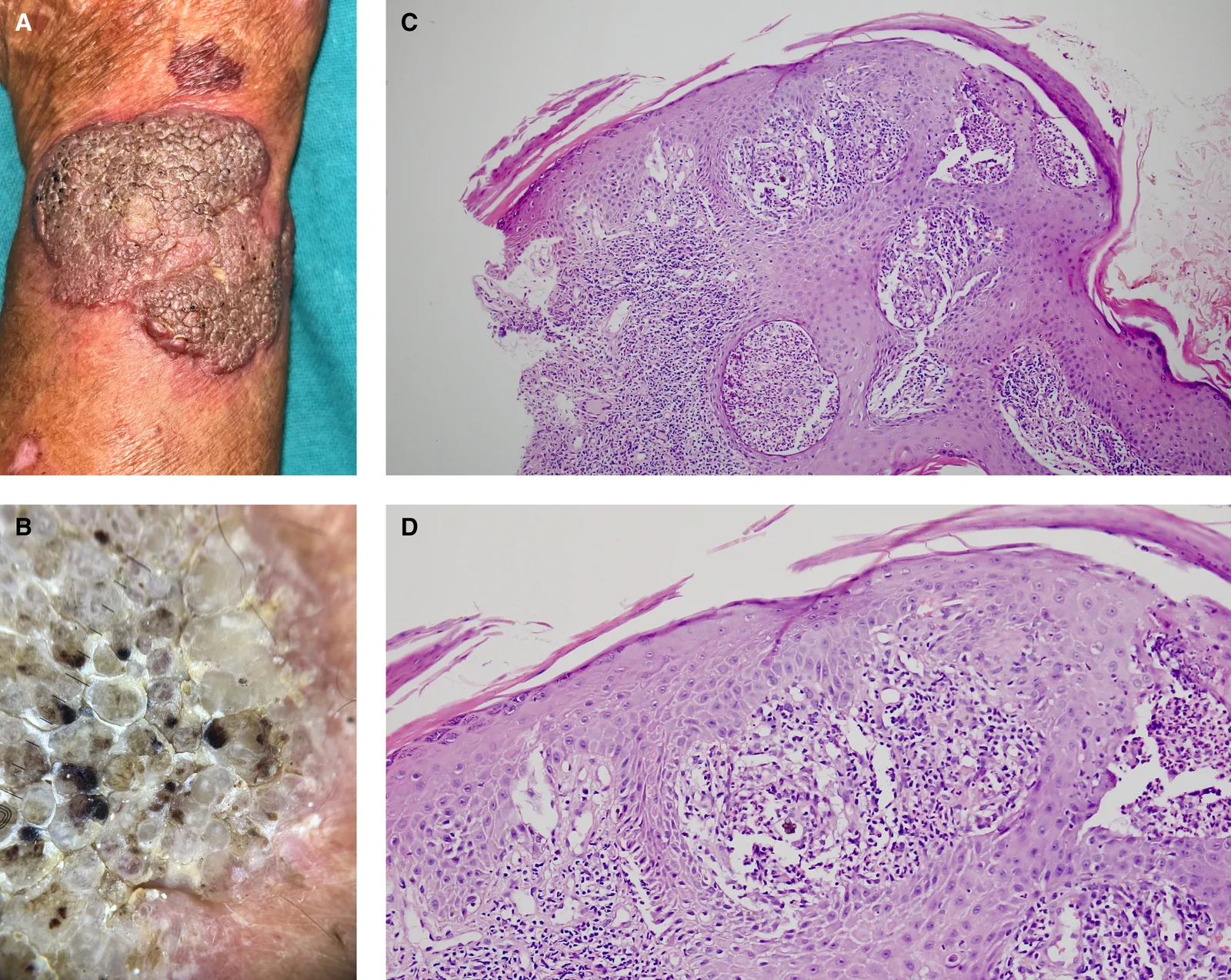
(**A**) A well-defined 5 × 8-cm hyperkeratotic plaque with a mamillated surface and a few areas showing the presence of black dots and yellowish crusting on the extensor aspect of the left forearm. (**B**) Dermoscopic examination revealed heavy keratinization with numerous clustered black–red dots across the surface corresponding to transepidermal elimination. (**C**) Histopathological examination revealed epidermal hyperkeratosis, parakeratosis, and pseudoepitheliomatous hyperplasia with dense inflammatory infiltrate composed of lymphocytes, neutrophils, and plasma cells with the formation of occasional suppurative granulomas; within these granulomas, clusters of rounded, pigmented, fungal profiles with copper penny appearance were noted (hematoxylin and eosin, 40×). (**D**) Hematoxylin and eosin, 100×.

Chromoblastomycosis is a subcutaneous mycosis caused by many fungi, mainly *Fonsecaea*, *Cladophilophora*, *Phialophorae*, etc. Trauma is the principal mode of infection. The differential diagnoses of the classic form include infective causes, such as lupus vulgaris,^1^ localized cutaneous leishmaniasis,^2^ and sporotrichosis,^3^ and noninfective causes, such as verrucous discoid lupus erythematosus and squamous cell carcinoma^4^ in some cases. Tuberculosis verrucosa cutis usually presents as a scaly verrucous plaque, and it typically lacks black dots; dermoscopy shows diffuse hyperkeratosis without the clustered black–red dots of transepidermal elimination with orange–yellow areas, and histopathology shows caseating epithelioid granulomas, occasionally with demonstrable acid-fast bacilli. Fixed cutaneous sporotrichosis often presents as a solitary verrucous plaque with surface ulceration or crusting at the site of trauma, with histopathology showing suppurative granulomas and occasional asteroid bodies but no muriform cells. Culture yields cigar-shaped budding yeasts of sporothrix rather than melanized sclerotic bodies. In chromoblastomycosis, the demonstration of copper-penny muriform bodies on histopathology (supported by culture) and the dermoscopic clustered black–red dots are the decisive distinguishing features. Morphological types of chromoblastomycosis include nodular, tumorous, verrucous, plaque type, and cicatricial type.^5^ The presence of black dots, which represent the transepithelial elimination of fungal colonies, is the most reliable dermatoscopic sign to aid in diagnosis. Potassium hydroxide mount examination can also aid in diagnosis by revealing the sclerotic bodies, although the sensitivity is quite low. Once suspected, histopathological examination, fungal culture, and molecular diagnostic methods should be used for confirmation of diagnosis. Chromoblastomycosis often requires prolonged therapy with systemic antifungal agents, such as itraconazole or terbinafine, sometimes in combination with physical modalities, including cryotherapy, thermotherapy, or surgical excision.^5^ Early diagnosis and prompt initiation of treatment are crucial to prevent progressive fibrosis, disability, and disfiguring scars, particularly at cosmetically sensitive and functionally important sites, where destructive treatment modalities may themselves contribute to scarring and deformity. Infectious granulomatous diseases, such as lupus vulgaris, cutaneous leishmaniasis, sporotrichosis, and chromoblastomycosis, may be clinically difficult to differentiate, highlighting the need for rapid point-of-care diagnostic tests. However, the presence of characteristic black dots and dermoscopic findings of clustered black–red dots corresponding to transepidermal elimination can serve as valuable bedside clues favoring chromoblastomycosis, whereas histopathological examination acts as the confirmatory test.
